# Macrosystem community change in lake phytoplankton and its implications for diversity and function

**DOI:** 10.1111/geb.13626

**Published:** 2022-12-21

**Authors:** Benjamin Weigel, Niina Kotamäki, Olli Malve, Kristiina Vuorio, Otso Ovaskainen

**Affiliations:** ^1^ Research Centre for Ecological Change, Organismal and Evolutionary Biology Research Programme, Faculty of Biological and Environmental Sciences University of Helsinki Helsinki Finland; ^2^ Finnish Environment Institute Jyväskylä Finland; ^3^ Finnish Environment Institute Helsinki Finland; ^4^ Centre for Biodiversity Dynamics, Department of Biology Norwegian University of Science and Technology Trondheim Norway; ^5^ Department of Biological and Environmental Science University of Jyväskylä Jyväskylä Finland

**Keywords:** biodiversity, community assembly, freshwater, HMSC, lake, niche conservatism, species distribution modelling, traits

## Abstract

**Aim:**

We use lake phytoplankton community data to quantify the spatio‐temporal and scale‐dependent impacts of eutrophication, land‐use and climate change on species niches and community assembly processes while accounting for species traits and phylogenetic constraints.

**Location:**

Finland.

**Time period:**

1977–2017.

**Major taxa:**

Phytoplankton.

**Methods:**

We use hierarchical modelling of species communities (HMSC) to model metacommunity trajectories at 853 lakes over four decades of environmental change, including a hierarchical spatial structure to account for scale‐dependent processes. Using a “region of common profile” approach, we evaluate compositional changes of species communities and trait profiles and investigate their temporal development.

**Results:**

We demonstrate the emergence of novel and widespread community composition clusters in previously more compositionally homogeneous communities, with cluster‐specific community trait profiles, indicating functional differences. A strong phylogenetic signal of species responses to the environment implies similar responses among closely related taxa. Community cluster‐specific species prevalence indicates lower taxonomic dispersion within the current dominant clusters compared with the historically dominant cluster and an overall higher prevalence of smaller species sizes within communities. Our findings denote profound spatio‐temporal structuring of species co‐occurrence patterns and highlight functional differences of lake phytoplankton communities.

**Main conclusions:**

Diverging community trajectories have led to a nationwide reshuffling of lake phytoplankton communities. At regional and national scales, lakes are not single entities but metacommunity hubs in an interconnected waterscape. The assembly mechanisms of phytoplankton communities are strongly structured by spatio‐temporal dynamics, which have led to novel community types, but only a minor part of this reshuffling could be linked to temporal environmental change.

## 1 INTRODUCTION

Freshwater ecosystems harbour unique biodiversity, are vital for human well‐being and provide a plethora of crucial ecological and socio‐economic services (Dudgeon et al., 2006; Falkenmark, 2003; Myers, 2003). However, freshwater biodiversity is declining globally (IPBES, 2019), and lakes are among the aquatic ecosystems most vulnerable to anthropogenic pressures (Mammides, 2020). At the terrestrial–aquatic interface, lake communities are directly influenced by both aquatic drivers and impacts stemming from terrestrial activities, such as nutrient loadings from agriculture, soil composition from adjacent forests and fields, and urban areas in the lake catchment (Dudgeon et al., 2006). As primary producers, phytoplankton communities are directly and, owing to their short generation time, rapidly affected by such impacts. Hence, they are frequently used as indicators to assess the ecological status (i.e., water quality) of aquatic systems (e.g., European Comission, 2000; Lepistö et al., 2004; Padisák et al., 2006). Recognizing lakes as sentinels for environmental impacts (Williamson et al., 2008), it is paramount to have a profound understanding of how species communities in lakes, such as phytoplankton, respond to anthropogenic impacts at multiple spatial and temporal scales.

Although lakes are often studied as closed and isolated environments at individual or local scales, they are often part of a network connected within watersheds and river basins (Heino et al., 2020; Soranno et al., 1999), enabling species dispersal and hierarchically structured impacts of environmental changes. It is well known that community dynamics and species diversity are scale dependent (Chase et al., 2019; Chase & Leibold, 2002; Jarzyna & Jetz, 2018) and contingent on mechanisms such as competitive interactions, niche differentiation and environmental filtering (Hardin, 1960; Keddy, 1992; Mayfield & Levine, 2010). Recent research has highlighted lakes as meta‐ecosystems at the waterscape level to account for these processes at different spatial scales, taking a macro‐ecological approach (Heino et al., 2020). Such scale‐related aspects in metacommunity frameworks are more frequently studied in terrestrial ecosystems, yet with an increasing focus also in the aquatic realm (Hortal et al., 2014). A growing body of literature now focuses on phytoplankton community assembly processes and the contributing abiotic and biotic interactions, while also considering the role of species traits (García‐Girón et al., 2020; Guo et al., 2019; Klais et al., 2017; Loewen et al., 2021; Mo et al., 2021). One of the most pervasive traits is size, because it propagates various associated physiological properties that drive community structure and functioning (Andersen et al., 2016; Hillebrand et al., 2022). The globally declining size structure of phytoplankton has previously been linked to warming conditions in both marine and freshwater systems (Finkel et al., 2010; Zohary et al., 2021), pointing to functional reorganizations under proceeding impacts of climate change. Approaches for understanding community dynamics that include information on functional and evolutionary characteristics of species, such as traits or phylogenetic relationships, are useful when investigating impacts on ecosystems, their biodiversity and ecosystem function. Trait‐based approaches have already proved beneficial in the assembly rule framework (Cadotte et al., 2015; McGill et al., 2006), because traits can explain species responses to environmental gradients from a more mechanistic point of view (Edwards et al., 2013; Gagic et al., 2015). Some traits can be good predictors for species occurrences considering, for example, their environmental niche, resource competition or dispersal, whereas other traits can be more relevant in the light of ecosystem services and water quality management, such as the toxicity of occurring species (Grizzetti et al., 2019). However, certain traits can be linked not only to species that have undergone environmental filtering, but also to evolutionary constraints of closely related species that are likely to share similar traits and occurrence patterns. This phenomenon is known as phylogenetic niche conservatism (Harvey & Purvis, 1991). Community responses are determined by species‐specific abilities to cope with environmental conditions (e.g., the width of niche space and adaptability), and by the ability to coexist with other species in terms of resource availability/competition, which is constrained to varying degrees by niche conservatism (Wiens et al., 2010).

During recent decades, joint species distribution modelling has become a valuable tool in global change ecology to gain a better understanding of community‐wide responses to changing environments (Ovaskainen & Abrego, 2020). An advantage is that species within a community are not modelled independently but with an underling joint structure that accounts for abiotic and biotic filtering simultaneously. Most studies either model community responses to environmental change or trait–environment relationships based on empirical data using separate ordination methods, and only few studies consider responses of species to environmental variation that share a joint structure, depending simultaneously on species traits and phylogenetic relationships.

Here, we leverage an extensive phytoplankton community data set, including locations at 853 lakes spanning the whole country of Finland and sampled over four decades (1977–2017). We model species community composition to investigate environmental filtering by quantifying species‐specific variance partitioning of environmental covariates and determine species co‐occurrence patterns at different hierarchical spatial and temporal levels. To highlight emerging differences in community composition over space and time, we use a clustering algorithm to uncover different regions of common community profile (i.e., community types sharing similar species compositions). We also account for phylogenetic constraints and incorporate six traits, reflective of resource competition, physiology, morphology and the ability to produce toxins, to investigate how the functional composition of emerging community types has changed over time and how traits propagate species responses to environmental conditions.

We hypothesize that environmental filtering over four decades has led to niche separations following the species sorting paradigm in metacommunity ecology (Leibold et al., 2004), restructuring community compositions and resulting in a high prevalence of tolerant species and a low prevalence among remaining species within genera (i.e., taxonomic dispersion).

Exploring the relationship between regions of common community profiles and diversity metrics, at taxonomic and trait levels, enables us to understand how environmental impacts influence aspects of phytoplankton biodiversity and function on a macrosystem scale (*sensu* Heffernan et al., 2014).

## 2 MATERIALS AND METHODS

### 2.1 Study area and monitoring

Our study area includes 853 boreal lakes in Finland, spanning a ~1200 km latitudinal gradient. We included phytoplankton community data from the Finnish national phytoplankton monitoring database. This database is maintained by the Finnish Environment Institute (SYKE) and comprises nationwide phytoplankton community data from lake surface water samples taken during the summer months (July and August) between 1977 and 2017 (open data portal: http://www.syke.fi/en‐US/Open_information). All phytoplankton samples were preserved with acid Lugol's solution and analysed using the standard Utermöhl technique (CEN, 2006).

All lakes were not monitored each year, with the majority being monitored sporadically over the past four decades. Large lakes were commonly monitored at multiple spatially distant sites. Hence, we included 853 lakes, but the number of spatially distinct sampling sites in our study was 1057. To investigate scale‐dependent impacts on communities, we considered an increasing hierarchical spatial scale from the local level of sample site (*n* = 1057) nested in watershed (*n* = 666), nested in the regional level of river basin (*n* = 54). Watersheds are considered to be lake catchment areas (i.e., the area of land that catches rain or snow and drains into the lake), whereas river basins comprise many watersheds that jointly drain to coastal areas.

### 2.2 Data acquisition

**FIGURE 1:**
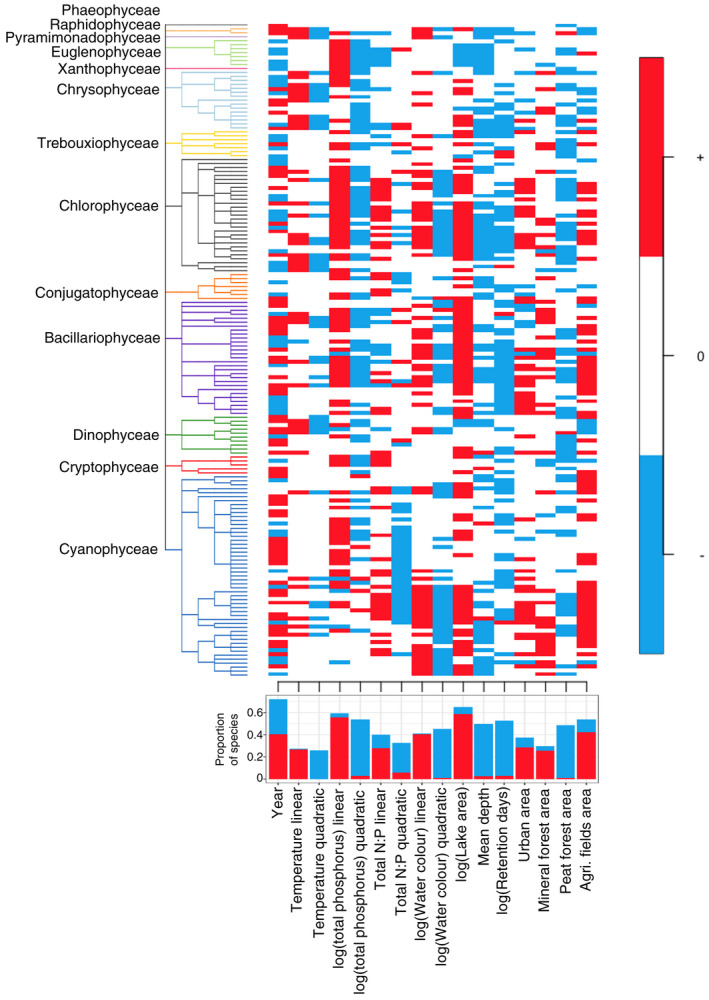
Phylogenetically structured responses of species occurrences to the covariates. Responses that are positive with ≥95% posterior probability are shown in red. Responses that are negative with ≥95% posterior probability are shown in blue. Responses that did not gain strong statistical support are shown in white. The bar plot at the bottom of the figure summarizes the proportion of species responses that are positive or negative for each covariate.

The Finnish monitoring database comprised 1043 species records. To avoid computational constraints for the joint species distribution model owing to the vast number of species and considering that most species are rare and therefore difficult to predict, we focused on the most representative species in the observed communities. For this, we selected those species that contributed to the cumulative upper 95% of the total biomass during the four decades of monitoring. This resulted in a total of 165 species belonging to 13 classes (Figure 1).

We included a suit of environmental predictors that are known to influence phytoplankton communities, ranging from physico‐chemical water variables to aspects of bathymetry and land use (Table 1). We included water temperature, in addition to nutrient concentrations, specifically total phosphorus and the ratio of total nitrogen to total phosphorus, and water colour. Covariates related to bathymetry were lake‐specific surface area, mean depth and retention days (the residence time of water in a lake). As proxies for the extent of land use around lakes, we included the proportions of urban area, agriculture fields and forests in the catchment of the lakes, considering mineral and peat soil types of forests separately, owing to their different properties in influencing runoff (Röman et al., 2018). All physico‐chemical water variables were recorded at the same time and location as the surface water sample for the species acquisition.

**TABLE 1:** Included environmental covariates and their relevance for structuring phytoplankton communities

Covariate group	Covariate	Unit	Relevance
Physico‐chemical	Water temperature	Degrees Celsius	Temperature directly affects metabolic and growth rates (Padfield et al., 2016) and bloom dynamics (Peeters et al., 2007)
	Total phosphorus (PTOT) concentration	Micrograms per litre	Phosphorus is a key component determining ecological quality and community structure in lakes (Schindler, 1977; Vitousek & Howarth, 1991), being a major driver of phytoplankton biomass (Phillips et al., 2008) and decreasing biodiversity and water clarity (Carpenter et al., 1998; Jeppesen et al., 2009)
	Total nitrogen to phosphorus (N:P) ratio	Unitless ratio	Proxy for nutrient availability. The N:P ratio describes one major factor regulating the dominance of planktonic communities by blue‐green or green microorganisms, with a decreasing N:P ratio favouring cyanobacterial blooming (Levich, 1996)
	Water colour	Milligrams of platinum per litre	Acts as measure for humic substances, dissolved organic matter and light attenuation (Håkanson, 1993; Nürnberg, 1998)
Bathymetry	Lake area	Square kilometres	Lakes can be considered as islands (Dodson, 1992) with a terrestrial surrounding, following the species area theory. Nutrient enrichment effects and phytoplankton diversity are dependent on ecosystem size (Baho et al., 2017)
	Mean lake depth	Metres	Lake depth integrates many other habitat features, such as water layer mixing, extent of the photic zone and nutrient distribution, and is known to impact phytoplankton functional diversity (Longhi & Beisner, 2010)
	Retention days	Days	Retention time influences many aspects of phytoplankton populations (Elliott et al., 2009). For example, the rate of water movement through a lake can contribute to the loss and gain of nutrients, organic matter and turbidity (Søballe & Kimmel, 1987)
Land use	Urban (or constructed) area	Percentage of catchment area	Catchment characteristics reflect the degree of anthropogenic pressures and sources of nutrients and runoff (Röman et al., 2018)
	Area of agricultural fields	Percentage of catchment area	
	Mineral forest area	Percentage of catchment area	
	Peat forest area	Percentage of catchment area	

To account for traits in species responses to environmental covariates, we included six traits characterizing important ecological aspects of phytoplankton after Weithoff (2003) and Litchman and Klausmeier (2008), namely, cell volume, nitrogen fixation ability, demand for silica, motility, chain forming ability and the ability to produce toxins, hereafter referred to as “toxic” (Table 2). The trait data matrix was acquired by the database curator, Dr Vuorio, and is available in the Supporting Information (Table S1).

**TABLE 2:** Included phytoplankton traits and presumed reflective ecological relevance

Trait	Trait category	Relevance
Cell volume	Continuous (in micrometres cubed)	According to allometric theory, serves as a determinant of physiological activities, such as growth and nutrient uptake (Weithoff et al., 2015)
Nitrogen fixation	Categorical (yes/no)	The ability of nitrogen fixation provides competitive advantages under nitrogen limitation (Flores, 2008)
Demand for silica	Categorical (yes/no)	Silica increases the specific weight, leading to higher sedimentation rates (Weithoff et al., 2015). Besides diatoms that need silica for their frustules, other orders require it for statospores, bristles and scales. Silicate bristles and spikes also contribute to grazing defence (Hamm et al., 2003)
Motility	Categorical (yes/no)	Mobile organisms can migrate into favourable conditions and impede sedimentation (Weithoff et al., 2015). Motility can also affect nutrient availability, with cell movements diffusing the boundary layer for nutrients around the cells (Pasciak & Gavis, 1974)
Chain forming	Categorical (yes/no)	Larger cell sizes generally experience lower grazing pressure. Hence, the formation of chains, which can be induced by biotic and abiotic factors, can result in reduced mortality by grazing pressure (Pančić & Kiørboe, 2018)
Toxicity	Categorical (yes/no)	Toxic phytoplankton species can develop harmful algae blooms (HABs). Such blooms have an impact not only on water quality, but also on species diversity, community structure and ecosystem functioning. Most HABs lead to a significantly decreased diversity and an impairment of many ecosystem functions (Litchman et al., 2010). Here, all toxic species belong to the class Cyanophyceae and are those able to produce cyanotoxins, including neuro‐ and hepatotoxins

In cases where there were no species‐specific trait data available, we applied the values of the closest taxonomic level for categorical traits and the mean values of the family level for the continuous cell volume (Weigel et al., 2016).

We also accounted for phylogenetic constraints in species responses to the environment by including the taxonomic relationships of species. Given that true phylogenetic information for species was not available, we used the *as.phylo* function in the *ape* package (v.5.5) (Paradis & Schliep, 2019) to build a phylogenetic tree based on the taxonomic levels of class, order, family, genus and species, and assuming a branch length of one between each node. We note that the assumption of equal branch length is not necessarily correlated with the times since species have diverged from their common ancestors, but we considered this approach to yield a reasonable proxy for species relatedness.

Related to the long‐term nature of the data, spanning four decades, there have been taxonomic changes of species identities stemming from improved identification protocols, keys and the variable identification skills and efforts of phytoplankton analysts. To account for these changes over time, potentially affecting inference on the emergence of new community compositions, we ran a conservative sensitivity analysis including only those species identities that have been classified consistently since the onset of the monitoring campaign. Thus, in cases where, for example, a previously identified species (*Aa*) was split into multiple species (*Aa*, *Ab*, *Ac*, …) over the course of the study time frame, we converged all subsequent emerging species *Ab*, *Ac*, … back to the original species, *Aa*, resulting in a total of 133 species instead of 165 (Supporting Information Supplementary material S1; Table S2).

### 2.3 Statistical analysis

We analysed the data with hierarchical modelling of species communities (HMSC; Ovaskainen et al., 2017; Ovaskainen & Abrego, 2020). HMSC belongs to the class of joint species distribution models (Warton et al., 2015), including a hierarchical layer for how species responses to environmental covariates depend on species traits and phylogenetic relationships (Abrego et al., 2017). Here we use spatially structured latent variables that were proposed originally by Ovaskainen et al. (2016) and later expanded to big spatial data (Tikhonov, Duan, et al., 2020). For the analyses, we used phytoplankton community data comprising the presence/absence of 165 species surveyed at 1057 sampling locations in 853 lakes (with some large lakes having more than one sampling site) across Finland over 40 years from 1977 to 2017, resulting in a total of 3774 spatio‐temporally explicit locations considering the repeated surveys over time. As sampling unit, we used the individual samples taken at each location. Given that we modelled presence/absence data, we applied a probit regression model.

As fixed effects, we included the reported physico‐chemical water covariates, aspects of lake bathymetry and land use (Table 1). Before the analysis, we ln‐transformed total phosphorous concentrations, water colour, lake area and retention days to achieve a more homogeneous distribution of the variables. To account for temporal trends in community trajectories not explained by the included covariates, we also added year as a linear fixed effect (Peltonen & Weigel, 2022). We assumed that species niches might include their optimum at intermediate values of physico‐chemical variables, hence we implemented these covariates as quadratic response functions (Antão et al., 2022; Weigel et al., 2021), whereas the remaining covariates were assumed to have a linear relationship. To account for the spatial nature of the study design and to investigate the scale‐dependent aspects of species co‐occurrence patterns, we included random effects at three spatial scales: site, nested in watershed, nested in river basin. The random effect of site was assumed to be spatially explicit and was implemented through the computationally efficient predictive Gaussian process for big spatial data (Tikhonov, Duan, et al., 2020). To account for the temporal stochasticity of the data, we also included year as random effect (Peltonen & Weigel, 2022). HMSC involves a hierarchical structure, examining how species responses to environmental covariates depend on species traits and phylogenetic relationships. Hence, we included traits (Table 2) and the described phylogenetic relationship of the species as a taxonomic tree in our model. The phylogenetic correlation parameter is denoted as ρ, with possible values between zero and one indicating whether the residual (after accounting for their traits) variance among the species environmental responses is independent (zero) or whether their environmental responses are fully structured by their phylogeny (one).

The community model described above examines the extent to which physico‐chemical water variables explain community variation. To examine how physico‐chemical water variables have changed over time, we built a second HMSC model, henceforth called the environmental model. The environmental model followed the same structure as the community model, but with physico‐chemical water variables as responses. We included the same predictors as for the community model, except for the physico‐chemical variables bathymetry, land use and year as fixed effects, and spatial and temporal random effects. This allowed us to quantify the contributions of year (temporal change), land use and lake bathymetry on the development of the physico‐chemical water variables while accounting for the hierarchical spatial structure of the data.

We fitted both the community model and the environmental model with the R package *Hmsc* (v.3.0.12) (Tikhonov, Opedal, et al., 2020), assuming the default prior distribution (see chapter 8 of Ovaskainen & Abrego, 2020). We sampled the posterior distribution with four Markov chain Monte Carlo (MCMC) chains, each of which was run for 37,500 iterations, of which 12,500 were removed as burn‐in. The chains were thinned by 100 to yield 250 posterior samples per chain, resulting in 1000 posterior samples in total. We assessed MCMC convergence by examining the potential scale reduction factors (Gelman & Rubin, 1992) of the model parameters [i.e., the beta (species–environment relationship) and gamma (trait–environment relationship) parameters; Ovaskainen et al., 2017].

We examined the explanatory power of the community model through species‐specific area under the curve (AUC) (Pearce & Ferrier, 2000) and Tjur's *R* ^2^ (Tjur, 2009) values, both of which measured how well the model was capable of discriminating between presences and absences. For the environmental model, we evaluated the explanatory power with *R* ^2^. We then followed Ovaskainen et al. (2017) to partition the explained variation among the fixed and random effects.

To illustrate the changes in community composition over space and time, we calculated regions of common profile based on the predicted species occurrence matrix at the sample level. We used the *NbClust* package (v.3.0) (Charrad et al., 2014) to determine an optimal clustering scheme, between two and ten clusters, using “method” = *kmeans* and “distance” = *euclidean*. *NbClust* delivers a framework for determining the best number of clusters by providing 30 indices and proposing the best clustering scheme based on the majority rule (i.e., the number of clusters that most of 30 indices agreed on). We investigated characteristics of the different clusters in terms of species prevalence, species richness and community‐weighted traits, in addition to the temporal progression of the physico‐chemical variables. We then assessed differences in species richness and the community‐weighted mean traits between community profiles, in addition to the differences in physico‐chemical variables at regions of common profile (RCPs). Given that homogeneity of variance was not given among RCPs, we used Games‐Howell post hoc test from the “*rstatix*” package v.0.7.0 (Kassambara, 2021), a test used to compare all possible combinations of group differences when the assumption of homogeneity of variances is violated. All analysis was performed in R v.3.6.3 (R Development Core Team, 2020).

## 3 RESULTS

### 3.1 Model convergence and fit

**FIGURE 2:**
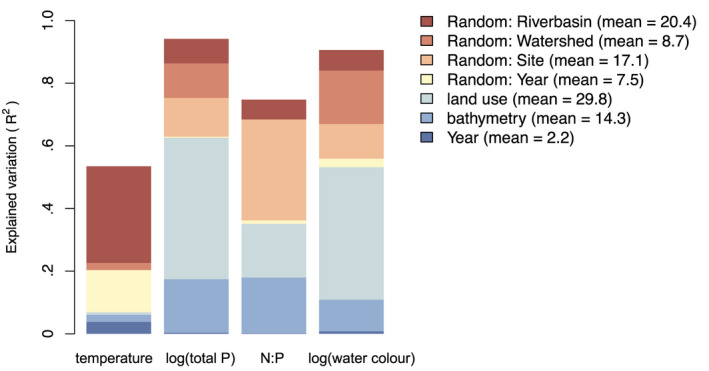
Explained variation (*R* ^2^) of physico‐chemical water variables in the environmental model. Variation is partitioned into responses to fixed and random effects. The bar plot shows physico‐chemical variable‐specific results, and the key includes the averages over all variables.

The MCMC convergence of both the community model and the environmental model was satisfactory. This was indicated by the potential scale reduction factors (psrf) of the model parameters being <<1.1. For the beta parameters, point estimates of the psrf were on average 1.03 [mean upper credible interval (CI) = 1.06] and the gamma parameters on average 1.003 (mean upper CI = 1.012). The community model showed a good fit to the data, with a mean AUC value of .88 and mean Tjur's *R* ^2^ value of .23 (Supporting Information Figure S1). The environmental model had a high explanatory power of *R* ^2^ = .78 (Figure 2).

### 3.2 Temporal change of water physico‐chemistry

We found that most of the variation in physico‐chemical water variables was of a spatial nature, and much related to land‐use covariates. In contrast, only a minor part of the variation was of a temporal nature, with a negligeable part being related to a systematic trend over time, whereas a larger part was attributed to year‐to‐year random variation (Figure 2). The linear trend was positive for temperature and water colour (posterior probability for positive effect 100%; Supporting Information Figure S2). Total phosphorus also followed a linear positive, but weaker trend (posterior probability for positive effect 86%; Supporting Information Figure S2), with minor absolute changes in comparison to temperature and water colour. The N:P ratio showed a negative linear trend (posterior probability for negative effect 80%; Supporting Information Figure S2).

### 3.3 Explained variation in species occurrences

We observed strong spatio‐temporal structuring at the species and community levels. About half of the explained variation was attributed to the spatially structured random effects of site identity (ID; 18.6.%), watershed (10.8%) and river basin (7%). The random effect of year was attributed 17.8% of explained variation. Among the fixed effects, total phosphorus explained most of the variation (13.8%), followed by the linear effect of year (10.7%). Given that none of the remaining fixed effects explained substantial amounts of variation (i.e., individually explained <5% variation; see Supporting Information Figure S3), we examined the proportions of variation explained by covariate groups [i.e., physico‐chemical water parameters (temperature, N:P ratio, total phosphorus and water colour), bathymetry (lake area, mean depth and retention days) and land use (urban area, agricultural field and forest type)]. The physico‐chemical water variables contributed the most to the partitioned variation (18%), followed by land use (9.4%) and bathymetry (7%). The species traits explained on average 6.7% of the among‐species variation in species responses to the fixed effects, with the strongest effects being related to species responses to water colour, water temperature and lake area (28.6, 27.2 and 21.4% of explained variation, respectively; Supporting Information Figure S4).

### 3.4 Phylogenetically structured species responses to covariates

Related species showed similar responses to environmental covariates (Figure 1), reflected in the posterior mean of the phylogenetic correlation parameter (ρ) being .84 (with a 95% CI from .76 to .91). The linear effect of year influenced most of the species occurrences (either negatively or positively) with ≥95% posterior probability. With this level of statistical support, temperature affected one‐fifth of the species occurrences, mostly with a positive linear response and a negative quadratic response. More than half of all species occurrences were affected by total phosphorus concentrations, mostly with a positive linear response and a negative quadratic response. Concerning aspects of bathymetry, almost two‐thirds of the species showed positive associations with total lake area, whereas retention days and lake depth had negative effects on about half of the species. Statistically supported responses to the size of urban areas and agricultural fields were mainly positive. When investigating the impact of different forest soil types in the catchment area, we found mineral forest soils to affect phytoplankton occurrences positively, whereas peat forest soils had a negative impact (Figure 1).

### 3.5 Spatial and temporal scales of species variation

**FIGURE 3:**
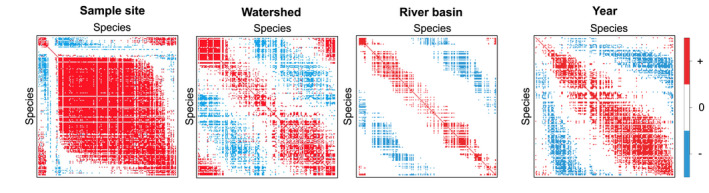
Residual association among species at spatial and temporal scales. Pairs of species illustrated by red and blue show positive and negative associations, respectively, with statistical support of ≥95% posterior probability. Residual associations here can be interpreted as species co‐occurrences after accounting for their environmental niche (i.e., they are species co‐occurring more or less often than by chance at the spatial or temporal scale after considering their environmental responses).

We found species residual co‐occurrences to display highly scale‐dependent patterns. At the local level of sampling site, a large proportion of species showed positive co‐occurrence (Figure 3). When increasing the spatial scale to the level of watershed and, in particular, to the level of river basin, the number of positive co‐occurrences decreased, and the number of negative co‐occurrences increased. At the level of year, we observed both positive and negative associations. Hence, we observed variation in species richness (as indicated by positive associations) over small spatial scales, whereas we observed variation in community composition (as indicated by negative and positive associations) over larger spatial scales and over the years.

### 3.6 Changes in taxonomic composition

**FIGURE 4:**
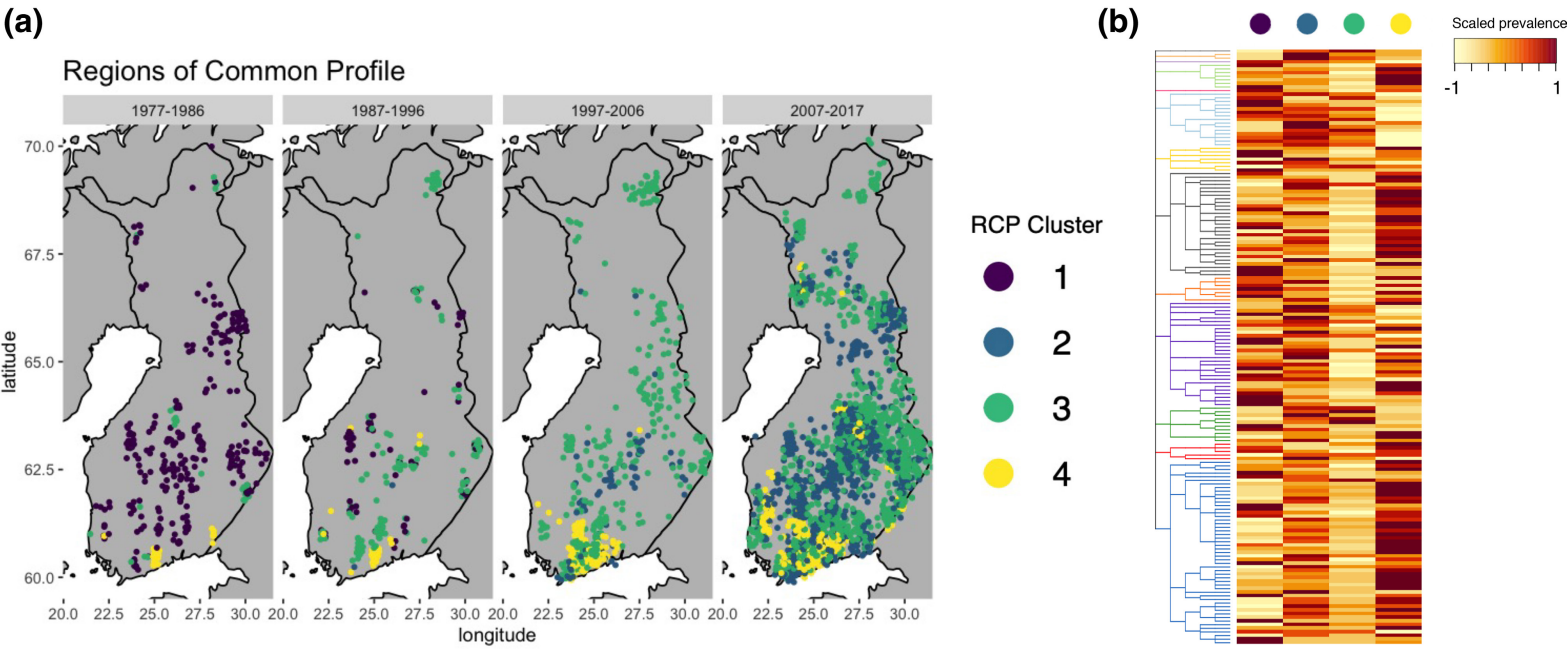
(a) Regions of common profile based on optimal clustering of predicted species compositions in lakes. Data are displayed in aggregations of 10 years to highlight the temporal aspect of change. To avoid overlapping data points, the data are plotted with a jitter of 0.2° for latitude and longitude. (b) Scaled prevalence of species in each region of common profile (RCP), following the phylogenetic structure of Figure 1.

Above, we reported how phytoplankton communities respond to variation in environmental conditions, identifying the total amount of phosphorus as a key driver of species responses. During the four decades spanned by our data, lake ecosystems have undergone major changes in community composition (Figure 4a), illustrated by changing RCPs. We highlight four emerging RCP clusters based on the predicted community composition at the level of sample ID. RCP cluster 1 was dominant during the mid‐1970s–1980s throughout Finland, but a complete restructuring took place, which resulted in RCP 1 disappearing after the mid‐1990s and a novel RCP cluster 2 and previously scarce cluster 3 becoming dominant, with RCP cluster 4 also becoming more common and expanding from only a few lakes in southern Finland northwards (Figure 4a).

**FIGURE 5:**
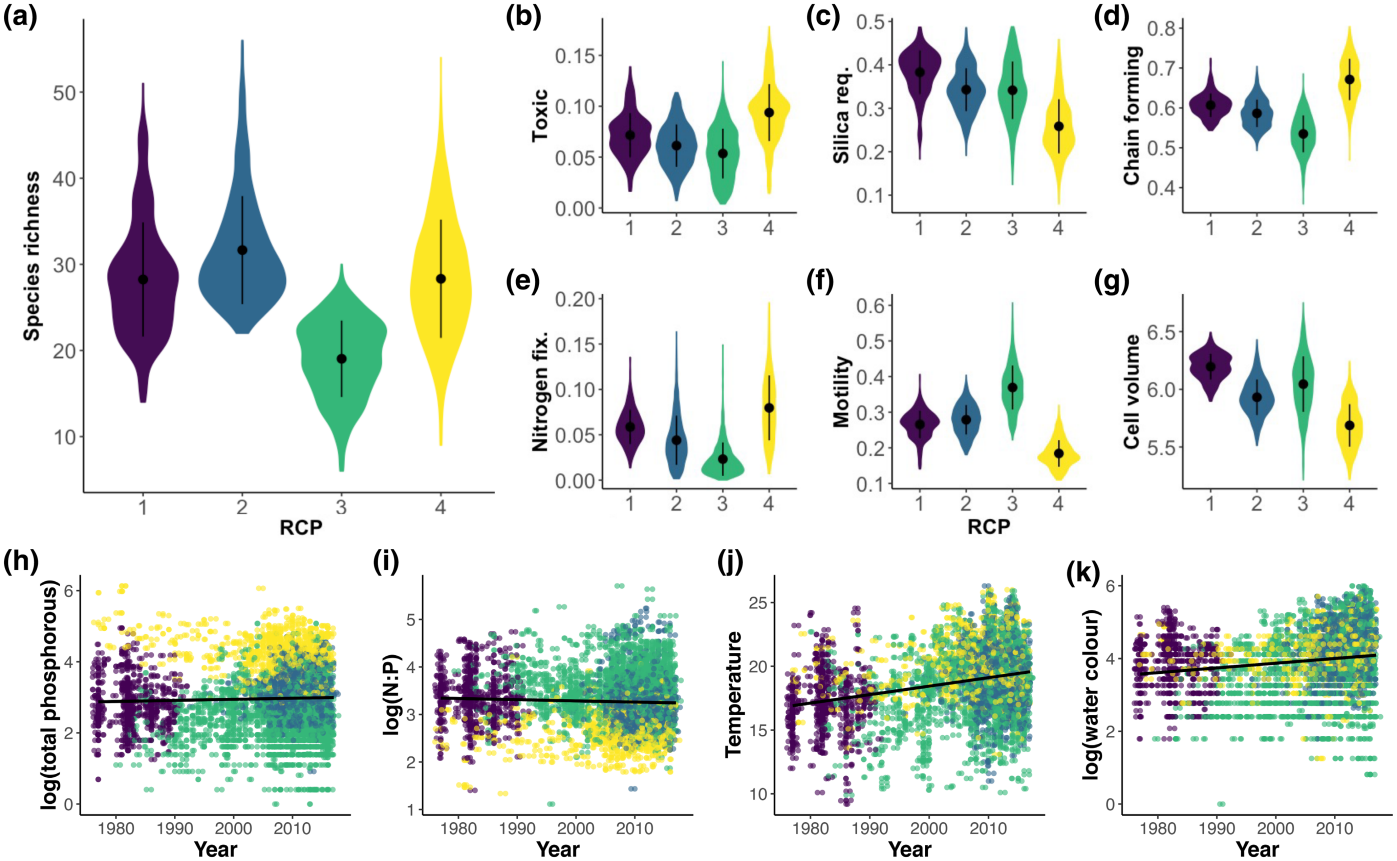
Illustration of community characteristics at the four regions of common profile (RCPs). (a) Species richness, (b–g) community‐weighted trait values and (h–k) environmental associations of RCPs over time. Colours of RCPs are the same as in Figure 3. The black regression lines illustrate the slope of environmental change over time.

Each RCP was associated with different prevalence patterns of species, of which some RCPs showed strong phylogenetic structuring (Figure 4b). RCP 1 showed a high prevalence of few dominant species within several taxonomic classes, whereas RCP 4 was characterized by a high prevalence of most species contained in the classes of Cyanophyceae (Cyanobacteria) and Chlorophyceae (green algae), in addition to Euglenophyceae and Cryptophyceae (both flagellated). RCP 2 showed no clear phylogenetic structuring in terms of prevalence but depicted overall relatively high species prevalence. This contrasted with RCP 3, which showed generally low prevalence of species, with the exception of Dinophyceae (dinoflagellates) and Chrysophyceae (golden algae). We found that mean species richness differed among all RCPs, with the exception of 1 and 4, with RCP 2 displaying the highest and RCP 3 the lowest richness (Figure 5a; Supporting Information Figure S5).

Linking the RCPs to environmental conditions, we found RCP‐specific environmental associations over the past 40 years (Figure 5h–k; Supporting Information Figure S6). RCP 4 was associated with higher total phosphorus concentrations compared with the remaining RCPs, whereas RCP 3 was especially prevalent in the lower phosphorus concentrations (Figures 5h; Supporting Information Figure S6a). RCPs 1 and 2, representing the originally dominant cluster and the novel cluster, respectively, were most prevalent in between the highest (RCP 4) and lowest (RCP 3) phosphorus contractions (Figure 5h), but with RCP 2 being found, on average, at higher phosphorus concentrations (Supporting Information Figure S6a). We found roughly the opposite pattern for RCPs for the N:P ratio (Figure 5i). Temperature increased over the study time frame (Figure 5j; Supporting Information Figure S2). RCP 4 was associated with the highest temperatures compared with the other RCPs, whereas RCP 1 was found at the lowest temperatures (Figure 5i; Supporting Information Figure S6c). Water colour has also increased over the past decades (Figure 5k; Supporting Information Figure S2). RCP 3 was present in the entire spectrum of water colours but was also the only RCP with high prevenances in clearer water (lower colour value), whereas RCP 2 communities were at the highest water colour values (i.e., darker waters; Figure 5k; Supporting Information Figure S6d).

Despite some changes in taxonomic identification procedures that affected 32 of the initial 165 species (mainly after the year 2000) our sensitivity analysis considering only the previous species complex of 133 species showed the same clustering results (Supporting Information Figure S7).

### 3.7 Change in community traits

The RCPs also differed by their community‐weighted traits. Comparing RCPs 1, 2 and 3, we found decreasing proportions of their potential toxicity (Figure 5b), silica requirements (with a non‐significant difference between RCPs 2 and 3; Figure 5c; Supporting Information Figure S5), chain‐forming (Figure 5d) and nitrogen‐fixating species (Figure 5e), in addition to a decrease in cell volume (Figure 5g). These decreasing trends also held true over time (Supporting Information Figure S5). In contrast, we found an increase in motile species from RCP 1–3 and over time (Figure 5f; Supporting Information Figure S5f). RCP 4 differed substantially from the other RCPs, with its toxicity, chain‐forming and nitrogen‐fixating traits being significantly higher and its requirement for silica, motility and cell volume being significantly lower than those in RCPs 1–3 (Figure 5b–g; Supporting Information Figure S5).

Additionally, we found strong relationships of the physico‐chemical variables with community‐weighted mean traits, when investigating their marginal effects. Temperature had a negative effect on silica requirement and cell volume (Supporting Information Figure S8a). Total phosphorous also showed negative effects on silica requirements and cell size but also on motility, while having a positive effect on the nitrogen‐fixating and chain‐forming traits (Figure S8b). The N:P ratio also had negative effects on silica requirement and cell size, while having a positive effect on the chain‐forming trait (Figure S8c). Water colour had a positive effect on silica requirements and a negative effect on motility (Figure S8d).

## 4 DISCUSSION

The concept of lakes as meta‐ecosystems helps to structure the large‐scale impacts of environmental change on biodiversity and ecosystem functions (Heino et al., 2020). Here, we have shown that a nationwide restructuring of lake phytoplankton communities took place in Finland over the past four decades, also resulting in altered community trait profiles. The historically most dominant and widespread community profile has vanished over time, now being replaced by two previously rarer and one novel community profile (Figure 4a). The emerging distribution of different RCPs results from deviating community compositions in a macrosystem spatio‐temporal context. However, we did not find strong evidence that the restructuring of communities can be explained by temporal change in the environment. Nutrient concentrations contributed much to the explained variation in species occurrences but changed only marginally over time. This suggests that the temporal change in the community profiles (i.e., the disappearance of RCP 1 and emergence of other community types) might be linked to changes in some unobserved environmental covariates or to processes other than environmental filtering, such as biotic interactions or stochastic processes. However, at macroecological scales, spatial variation of ecosystem properties might exceed temporal variation (Soranno et al., 2019), which is also reflected in our environmental model, in which spatial aspects tend to explain the variation of physico‐chemical water variables better than temporal aspects.

Despite the lack of strong evidence for temporal environmental filtering leading to the restructured communities, effects of land‐use and climate change have previously been linked to restructuring of taxonomically related population trends of communities, in addition to their function (Mouton et al., 2022). Here, we show that the interplay between environmental covariates and species occurrences (i.e., species niches) display a robust phylogenetic structure. Within these structured responses, species prevalence shifted from high taxonomic dispersion [i.e., only few highly prevalent (dominant) species per class] to low taxonomic dispersion (i.e., nearly all species with similar prevalence within classes), comparing the previously dominant with the more recent community profiles (Figure 4b). This contrasts with our assumption that over time, taxonomic dispersion (i.e., the prevalence of only a few tolerant species within genera and families) would increase under strong environmental filtering (Passy et al., 2017). Instead, we observed lower taxonomic dispersion, with overall more homogeneous, but community type‐specific species prevalence, which could be explained by a strong phylogenetic niche conservatism. This phylogenetic signal implies phylogenetically correlated response traits (not included in our analysis) that ultimately determine the species niche (Burner et al., 2021; Srivastava et al., 2012), also echoing that most phytoplankton traits are, to some degree, evolutionarily conserved (Bruggeman, 2011). We found a high prevalence especially of Cyanobacteria species in a more frequently appearing community profile (RCP 4), which is in line with globally observed trends of a higher prevalence of Cyanobacteria owing to the warming climate (Elliott et al., 2009; Kosten et al., 2012; Wagner & Adrian, 2011).

In our work, the included traits reflecting aspects of resource competition, physiology, morphology and toxicity did not explain major proportions of the variation among the species in their responses to the environmental covariates. However, distinct combinations of traits among species might support different responses to changes in the environment as a result of environmental filtering under altered niche spaces (Litchman et al., 2012; Mouillot et al., 2013). This is also reflected in the marginal effects of the physico‐chemical environment on the included traits (Supporting Information Figure S8). The differences in community‐weighted mean traits in relationship to ambient environmental conditions among the four RCPs indeed suggest altered community characteristics, hence, presumably, also functioning. Especially, the communities in RCP 4 differed from the rest (Figure 5). RCP 4 was found exclusively in environments with high total phosphorus concentrations, at low N:P ratios and at the highest temperatures over the study time frame (Figure 5h–j). In high‐phosphorus conditions, nitrogen‐fixating ability is beneficial for species, as indicated by the highest community‐weighted mean of nitrogen fixation (nfix trait) in this RCP, potentially giving the species complex a competitive advantage under nitrogen limitation. Furthermore, RCP 4 showed the lowest community‐weighted means for silica requirement, motility and cell volume, while displaying highest proportions of toxic and chain‐forming species. These ecological traits were specifically affected by the increase in temperature and phosphorus (Supporting Information Figure S8). All these characteristics point to a community dominated by Cyanobacteria (Cyanophyceae), which is also reflected in their high prevalence in this community cluster (Figure 4b). Surprisingly, despite finding strong support for environmental relationships of nitrogen fixation, chain formation and body size, especially with nutrient concentration, the effect on the trait “toxic” was not as strong (Supporting Information Figure S8). This might suggest that although Cyanobacteria became more prominent, especially in RCP 4, it might be proportionally more of those species with no ability to produce toxins. Yet, this functional group that can cause harmful algae blooms is well known for its lower nutritional quality and accessibility as food resource for zooplankton and other grazers, and therefore able to decouple trophic links and alter food web structure (Elser & Goldman, 1991; Wilson et al., 2006). However, in comparison to the historically dominant RCP 1, the emerging and currently dominant RCPs 2–4 also show, on average, smaller cell volumes and an overall decrease in the community‐weighted mean cell volumes over time (Figure 5g; Supporting Information Figure S5g). Smaller phytoplankton size structures have been linked to increased temperatures owing to climate change (Mousing et al., 2014; Winder et al., 2009; Zohary et al., 2021), which is also supported by our results from the marginal effect of temperature on size and might explain why the more recent community types are smaller compared with the historically dominant type of the late 1970s and 1980s. From the perspective of ecosystem function, this might become problematic because smaller phytoplankton are commonly favoured by smaller zooplankton grazers, altering the length of the food chain and resulting in less favourable resources for higher trophic levels (Litchman et al., 2015). Additionally, RCP 4, displaying the smallest sizes, is dominated by Cyanobacteria, which tend to be smaller but also less attractive or even detrimental to grazers owing to their lower nutritional value and toxins (Haney, 1987).

Here, we highlight both abiotic and biotic filtering processes of phytoplankton communities by modelling the species‐specific environmental drivers, through the implemented fixed effects, and aspects of biotic filtering, through the random effect part in our model. Although physico‐chemical water variables jointly contributed the most to the explained variation at the community level, the largest explained variation is related to spatial and temporal random effects. These random effects can be linked to biotic filtering (i.e., how the ecological interactions among species influence their occurrences, particularly their co‐occurrences; Figure 3), but also to missing random level‐specific covariates (Ovaskainen & Abrego, 2020). Considering the spatial scales from smallest (sample location) to largest (river basin), we found that the hierarchical spatial effects are decreasing in their importance in explaining species occurrences with increasing scale. This is in line with other findings (Heino et al., 2015; Ovaskainen et al., 2019) suggesting that connectivity and biotic filtering decrease towards larger scales. Thus, with increasing scales, local abiotic drivers are progressively governed by regional drivers, and the subsequent cross‐scale interactions among these drivers increase the macroscale complexity (Soranno et al., 2014). However, more frequent strong residual associations at smaller spatial scales might also suggest a lack of relevant environmental variables at smaller scales, hence there is more residual variation explaining species co‐occurrence patterns.

The relatively strong temporal signal, reflected here in the linear effect of year (fixed effect), suggests that there is a set of important unmeasured environmental parameters that have not been included in our study. These could be, for example, more detailed inorganic nutrient fractions, which have been shown to explain phytoplankton community properties better than including only total concentrations (Ptacnik et al., 2010; Trommer et al., 2020). Unfortunately, such detailed data are scarce, especially over broad spatio‐temporal scales. However, we found strong associations between the included environmental predictors and species occurrences that exceeded the explained variation of the linear temporal signal. Although temperature showed strong associations overall with about one‐third of the species, nutrient concentrations had an impact on almost twice as many species occurrences. When also considering aspects of land use in the catchment of the lakes, we found generally uniform responses among species, showing mainly positive associations with urban, mineral forest and agricultural areas, all being linked to higher nutrient inputs. However, peat forest area showed virtually only negative associations (Figure 1), which might be linked to more dissolved organic carbon in the runoff and the “browning” of lakes, hindering phytoplankton production through light absorption and shading (Klug, 2002; Thrane et al., 2014). Our results support this relationship further by displaying strong relationships with water colour as a proxy for water clarity. Dissolved organic carbon levels are increasing owing to climate change and land use (peat mining and forestry), an issue that has been neglected in water management until recently (Kritzberg et al., 2020). The lake water colour also affects the critical phosphorus threshold that triggers the growth of bloom‐forming Cyanobacteria (Vuorio et al., 2020) and decreases the nutritional quality of fish for human consumers (Taipale et al., 2016).

When taking the historically vastly dominating community type of RCP 1 as a baseline, we showed that environmental filtering and species sorting have resulted in a macrosystem‐wide restructuring of phytoplankton communities across Finland over the past four decades. This might potentially have wide‐reaching implications for lake ecosystem functioning, both for species interactions (e.g., food web structure) and for human well‐being (e.g., proportion of present species with the ability to produce toxins). Especially when considering the impacts of ongoing climate change and land‐use change, this might become a growing concern, fuelling temperature increase, elevated nutrient concentrations through runoff and the brownification of lakes, all of which are direct drivers of phytoplankton community structure. When considering lakes as metacommunity hubs in an interconnected waterscape, we show that the mechanisms of assembly of phytoplankton communities are structured by spatial and temporal dynamics, leading to new community types. Given that the establishment of such restructuring happens over several decades and can be showcased only on large spatial scales, it will be important to consider how different trajectories of environmental change might intensify the observed developments in community composition.

## AUTHOR CONTRIBUTIONS

B.W., N.K., O.M. and O.O. conceived the ideas and designed the methodology. K.V. curated and contributed the data. B.W. performed all analyses, including visualizations, and wrote the first draft of the manuscript. All authors contributed substantially to revisions and finalization of the manuscript.

## FUNDING INFORMATION

The study was funded by the Strategic Research Council of the Academy of Finland (Project 312650 BlueAdapt) and supported by Academy of Finland grants 309581 (to O.O.) and 311229 (to K.V.) and the European Research Council (ERC) under the European Union's Horizon 2020 research and innovation programme; grant agreement no. 856506; ERC‐synergy project LIFEPLAN (to O.O.).

## CONFLICT OF INTEREST

The authors declare no conflict of interest.

## BIOSKETCH

**Benjamin Weigel** is a postdoctoral researcher in the Research Centre for Ecological Change at the University of Helsinki. He is an aquatic community ecologist with a strong interest in long‐term ecological changes driven by anthropogenic pressures.

## Supporting information


Table S1:

## Data Availability

All data can be accessed via the link (https://doi.org/10.5061/dryad.h70rxwdnp). The provided files include all necessary data and a script to reproduce the presented results.
